# Locum physicians’ professional ethos: a qualitative interview study from Germany

**DOI:** 10.1186/s12913-018-3118-6

**Published:** 2018-05-08

**Authors:** Sabine Salloch, Birgit Apitzsch, Maximiliane Wilkesmann, Caroline Ruiner

**Affiliations:** 1 Institute of Ethics and History of Medicine, University Medicine Greifswald, Ellernholzstr. 1-2, 17487 Greifswald, Germany; 2 Sociological Research Institute (SOFI) Göttingen, 37085 Göttingen, Germany; 3 Faculty of Business, Economics, and Social Sciences, TU Dortmund University, 44221 Dortmund, Germany; 4 Institute of Work Science, Ruhr University Bochum, 44801 Bochum, Germany

**Keywords:** Locum physicians, Professionalism, Professional codes, Ethics, Germany, Qualitative methods

## Abstract

**Background:**

In contrast to other countries, the appearance of locum physicians as independent contractors constitutes a rather new phenomenon in the German health care system and emerged out of a growing economization and shortage of medical staff in the hospital sector. Locums are a special type of self-employed professionals who are only temporally embedded in organisational contexts of hospitals, and this might have consequences for their professional practice. Therefore, questions arise regarding how locums perceive their ethical duties as medical professionals.

**Methods:**

In this first qualitative study on German locum physicians, the locums’ own perspective is complemented by the viewpoint of permanently employed physician colleagues. Eighteen semi-structured interviews were conducted in 2014 to explore the professional practice of locum physicians from both groups’ perspectives with respect to doctor-patient-relationship, cooperation with colleagues and physicians’ role in society. The data were analysed using qualitative content analysis, including a deductive application and an inductive development of codes. The results were related to key tenets of medical professionalism with respect to the question: how far do locums fulfil their ethical duties towards patients, colleagues and the society?

**Results:**

The study indicates that although ethical requirements are met broadly, difficulties remain with respect to close doctor–patient contact and the sustainability of hiring locums as a remedy in times of staff shortage.

**Conclusions:**

Further qualitative and quantitative research on locum physicians’ professional practice, including patient perspectives and economic health care system analyses, is needed to better understand the ethical impact of hiring independent contractors in the hospital sector.

**Electronic supplementary material:**

The online version of this article (10.1186/s12913-018-3118-6) contains supplementary material, which is available to authorized users.

## Background

The German health care system has experienced considerable pressure in recent years due to a massive increase in health care costs and structural reforms in the direction of New Public Management [1, 2]. The nationwide introduction of a new form of reimbursement in the hospital sector replaced the old retrospective and procedural payment system in 2004. Diagnosis-Related Groups (DRGs) were introduced to increase the efficiency and the quality of care. However, this has also led to a shift of services towards outpatient facilities and an increasing intensity of treatment relative to the length of stay in hospital [3]. Growing competition between hospitals can be observed [4] accompanied by patient selection with respect to remunerative diagnoses [5]. In summary, the German health care system in the last decade is characterised by a shift from the provision of public health care to a quasi-market ideology.

The growing economisation in German health care has led to increased workloads and competition, which have had a negative impact on the physical and psychological well-being of health care staff [6, 7]. Particularly physicians who work in leadership positions in the hospital sector feel that their professional autonomy is threatened by misguided incentive systems which promote quantity instead of quality of care and can lead to a financially motivated broadening of medical indications [8]. In addition, the bureaucratisation of the medical job forms a major driving force for physicians leaving the patient care sector and seeking alternatives in other medicine-related branches or abroad [9]. These structural developments have a great impact on individual working conditions and prompted the emergence of locum physicians as a new type of freelancer on the German labour market scene. Whereas locum physicians already form a well-established type of self-employed workers in other countries [10], their appearance in German health care is a rather recent phenomenon.

Locum physicians can be defined as doctors who work temporarily for a contractually agreed fee in the in-patient or ambulatory sector without having their own practice or being permanently employed by a health care institution [11]. The great majority of German locums are medical specialists. They are characterised by diverse and complex professional biographies which often encompass stages of permanent employment alternating with independent contracting. There are about 4000 to 5000 locum physicians working part-time or full-time in Germany, which is equal to about 1% of all hospital physicians [12]. Their development is highly dynamic and depends on market forces as well as on changing regulations in professional law and employment restrictions. There has been a lack of research on German locum physicians to date. Previous quantitative studies have focused on the perspective of Human Resource Management [13] and atypical employment in health care [14]. An in-depth analysis of German locum physicians’ self-perception and their embedding in a complex working environment is due. In this article, we present major findings from the first qualitative interview study on German locum physicians.

As intermittent staff, locum physicians are hired by hospitals typically on a daily or weekly basis. They are, therefore, less embedded in organisational structures than the permanent employees. Locums enjoy a high degree of independence regarding their medical decision-making and overall performance, not least because of their professional status, and hospitals are highly dependent on the freelancers’ services. As locums do not form part of permanent health care teams, questions arise, for example, with regard to the character of their cooperation with physicians and non-physician colleagues. Related moral issues regard the individualistic orientations attached to independent contractors [15], or, more generally, the commitment of contingent workers [16]. Furthermore, the doctor–patient contact might be impaired by the short-time working periods of locum physicians. This qualitative interview study explores the impact of this new type of freelancing on the physicians’ ethical orientation with regard to patients, colleagues and their professional role in society. The locums’ own perspective stands in the main focus of this research. However, their view is complemented by interviews with permanently employed hospital physicians eliciting their perspectives on locums.

The data analysis refers to professional codes of ethics as a source of professional obligations. Medical professionalism is an intensively debated sociological concept and, at the same time, an influential value system which undergirds the self-understanding of the medical community. The traditional attributes of professionals are a systematic body of theory, professional authority and sanctions by the professional community [17, 18]. More recent contributions to the sociology of the profession stress the normative dimension of medical professionalism by highlighting the ethical duties in complex modern health care systems [19, 20]. Professional codes of ethics form a key component of professionalism. From early on, they provided the basis to stabilise a high quality of care and patient trust within physicians’ exclusive monopoly [21]. Nowadays, stressing professional autonomy and integrity is often used as a strategy to deal with factors such as new technologies, powerful market forces or globalisation which threaten the traditional ethos of physicians [22]. There are, however, certain controversies with respect to a contemporary understanding of professionalism which relate, for example, to the (seemingly) contradiction between professional and managerial principles [23]. Key tenets of professionalism such as patient welfare, cooperation with colleagues and maintaining an effective and stable health care system, still provide an appropriate background for discussing the ethical orientation of locum physicians.

## Methods

The qualitative study to investigate how locum physicians perceive their ethical duties towards patients, colleagues and society is based on 18 semi-structured interviews conducted face-to-face or on the telephone. These included 13 locum physicians and five permanently employed hospital physicians who were asked for their perspective on the locums’ professional behaviour. All interviewees were found through contacting gatekeepers, who have been known from former empirical studies in the context of hospitals conducted by the authors. Moreover, the authors posted calls in relevant newsgroups and made use of the snowballing technique. The team of authors conducted all interviews with duration of 45–90 min. The interviews were structured following an interview guide and covered interviewees’ biographies, professional behaviour and cooperation with core staff and superiors in hospitals, as well as the physician–patient relationship and critical incidents [24]. The interview guide in an abbreviated form can be accessed as a Additional file 1 to this article. All interviews, with an average duration of 49 min, were audio-recorded, transcribed and anonymised. The data analysis relied on qualitative content analysis [25], including an inductive development and a deductive application of codes. Starting from a theoretical formulation of definitions, for example, the consequences of engaging locum physicians, we applied these codes to the interview transcripts. National and international professional codes for physicians such as [20, 26] served as theoretical backgrounds with respect to standards for ethical behaviour in physician practice. We formulated inductive categories out of the material, for example, reasons for becoming self-employed, to be able to code relevant narratives. We then explicated coding rules for the categories and identified examples. The transcripts were encoded primarily individually and the codes were subsequently compared and discussed in several interdisciplinary team sessions. Correspondingly, the coding system was constantly checked and modified, inductively expanded and revised. After the revision of categories and coding agenda, we applied the final code scheme to all transcripts and interpreted the results. In this process, the data we gathered reached theoretical saturation and informational redundancy. Rater influence was controlled by having at least three researchers participate in the data interpretation process and by team discussions of the match of encoded codes to develop the code system jointly. In addition to the consensual validation, i.e. the authors carried out the interpretation jointly and in dialogue, the authors also guaranteed communicative validation, since they discussed their analysis and interpretation of findings with locum doctors as experts in the field of research (e.g. annual meetings of locum doctors).

## Results

The participants’ characteristics can be seen in Table 1. The subsequent presentation of data analysis is structured following the three target groups of physicians’ ethical duties, namely, the patients, colleagues and society.

- *Duties towards patients*

**Table 1:** Participant Characteristics

Participant Characteristics	
Locum physicians:	
Gender	10 male; 3 female
Age	40–73 y; median: 53 y
Work experience	11–35 y; median: 21 y
Clinical specialty	Anaesthesiology/A&E (6); Neurology/Psychiatry (3); Surgery (1); Urology (1); Gynaecology/Obstetrics (1); Radiology (1)
Permanently employed physicians:	
Gender	3 male; 2 female
Age	29–47 y; median: 31 y
Work experience	2–19 y; median: 6 y
Clinical specialty	Internal Medicine (3); Surgery (1); Gynaecology/Obstetrics (1)

The study with German locum physicians reveals that locums, as independent contractors in hospitals, declare a high level of orientation towards the patients’ well-being as a key component of ethical behaviour. They also indicate that they are in a better position than their permanently employed colleagues to promote patients’ welfare and autonomy due to their outsider role which allows them to overcome hierarchical constraints and financial imperatives related to the hospital’s economic situation when deciding on treatment options. Along these lines, locums regard themselves as being more patient-centred than permanently employed physicians.

"In fact, for me it is easier than for the permanently employed to carry on with the humane and patient-centred practice, which I have learned. They [the permanently employed physicians] are under a different pressure." (Locum Physician 03; 43).

Locum physicians stress that they can focus better on patient care instead of being overwhelmed by massive amounts of paperwork and other jobs which are perceived as “non-medical”. They feel that they can work more calmly and spend more time in the direct physician–patient encounter. In turn, they state that permanently employed physicians have to carry out the administrative work, because this kind of work has to be accomplished as part of their job.

From the perspective of the permanently employed physicians, however, the locums are in an equally unfavourable position with regard to the aim of a close patient contact and individualised care. They indicate that locums as intermittent staff are to the same degree confronted with a high work load, changing shifts and highly standardised procedures.

"When they were assigned for work during the day, they were usually hired for the whole week, and so their overall presence at the ward was as long as ours, and even when they were there only for nightshifts or for the weekend, their relationship to patients was no different from ours, because we also only have this one day, or the weekend [to work with the patient]." (Permanently Employed Physician 01; 39).

Furthermore, an extreme shortage of physicians can lead to patients’ expectations towards their doctors deflating to a minimum, so that they are not interested in the occupational status of their physician. Permanently employed physicians feel that locums are less able to estimate a patient’s clinical situation (and possible deterioration) if they only work on night shifts and do not observe the course of the disease daily. Additionally, the permanently employed physicians venture the guess that locums might not have a close personal relationship to patients, which might lead to an insufficient medical care.

"I can judge better about the severe illness of a patient who is feeling bad at night if I took care of him during the day as well …. And it is more difficult with patients who were looked after by colleagues, although I do care as much about them as about my own patients. But sometimes there were locum physicians who did not go to the patient who might have been suffering from pain at night." (Permanently Employed Physician 02; 39)

b) *Duties towards physician and non-physician colleagues*

In addition to an optimal quality of patient care, professionalism in the medical job is also mirrored in behaviour towards physician and non-physicians colleagues. Both aspects are inherently related, as patient care in the modern health care system is characterised by a fragmentation of knowledge through highly specialised subdisciplines and requires interdisciplinary teamwork in most areas. The interview study reveals that the working conditions of locums are characterised by constantly changing co-workers and surroundings, which necessitate a quick adaptation to formerly unknown institutional standards and health care teams. A beneficial relationship to junior and senior physician colleagues and to the nursing staff is a key condition for successful work. The cooperation between locums and permanently employed physicians is described predominantly by both groups of interviewees as being positive. Interview participants highlight the fact that locums are only hired in situations of an extreme staff shortage in which they serve as an urgently needed relief for the permanently employed staff.

"In my experience, the cooperation was very positive, because the hospitals only hire locum physicians when they no longer know how to get the work done otherwise. That means that the [permanently employed] colleagues had already ten on-call duties per month and are happy when someone comes who takes over some of the workload." (Locum Physician 04; 49).

The fact that locums are better paid for the same services than their permanently employed colleagues is raised in a number of interviews. The interviewees from both groups, however, do not see these income differences as a specific problem for the professional cooperation between permanent and intermittent staff:

"Some people were so money-grubbing that they became unappealing. This also happens with permanently employed staff, but in locums there’s more attention paid to that." (Locum Physician 05; 65).

Moreover, the permanently employed physicians stress the function of locums as educators. As the overwhelming majority of locums have the educational status of a medical specialist already, junior physicians profit from their colleagues’ advice when supervision from the regular superiors is missing.

"So we actually had quite positive experiences, because the benefit is that locum physicians are often specialists, and otherwise many colleagues are freshmen ... On the one hand, locum physicians are, of course, novices in terms of organisational structures, but, on the other hand, you can learn a lot ... that’s why I find it really positive." (Permanently Employed Physician 01; 31).

The key role of the nursing staff is discussed by the locum physicians especially with regard to their familiarity with organisational structures and routines. The constant claim of nursing staff regarding newly hired locums, however, can lead to tensions and to situations where the locums’ qualifications are doubted:

"It is really bad to be at a place you don’t know and where you always have to ask someone. Because then you always ask the nursing staff, and at some point the nurses say: “Hey, why did you hire this person who does not know his stuff”, or “Why did nobody tell him what to do?”" (Locum Physician 01; 237).

c) *Duties towards society*

Next to their actual behaviour towards patients and colleagues physicians in general fulfil an important role in society and contribute to the functioning of the health care system. The interviews reveal that locum physicians see themselves as members of the profession who understand the health care system better than others, including its deficits and incentive systems. Due to their self-employed status, they have experience with the internal structures of a large number of hospitals. As a form of “undercover agents” (Locum Physician 04; 167) they get to know about unfavourable working conditions, such as unpaid overtime and the physicians’ difficulties in fulfilling the requirements to become a medical specialist.

The interviewees estimate the impact of hiring locum physicians on the institution and the health care system ambivalently. While the locums benefit from the staff shortage which allows them to work flexibly in different contexts and to earn good incomes, the impact on the hospitals is estimated as being rather negative:

"From the perspective of the locum physician, I think it is good, but from the perspective of the hospital, it is not so good. I think they should employ more physicians when there is a shortage, because this [hiring locum physicians] is too expensive. It is ok as an emergency solution, but not permanently, it is not economic and not good." (Permanently Employed Physician 04; 37).

From the interviewees’ point of view, hiring locum physicians, therefore, does not contribute to a sustainable solution to deal with the physician shortage in the German health care system. Locums are seen instead as a stopgap for mastering the most urgent situations, which is, however, not the best option for securing high-quality health care provision for society.

## Discussion

In addition to financial gain, the execution of professional autonomy forms the main driver for German locum physicians to opt out of the system of permanent employment [27]. Locums perceive that they enjoy a high degree of freedom in their medical decision-making which takes place widely outside hospital hierarchies and organisational restraints. Whereas this aspect of professional practice is also mirrored in all interviews, the study delivers a diverse picture regarding the ethical duties which form a key component of medical professionalism. In eliciting the views of locums and complementing them with permanently employed physicians, the study shows that certain criteria, such as fruitful cooperation with physician colleagues, are successfully realised in hospital practice. At the same time, several concerns with regard to the ethical duties remain: Regarding the question whether locums are committed to the welfare of individual patients to the same degree, there are different estimations to be found between locum and permanently employed physicians. Moreover, the cooperation between locums and nursing staff is strained by the locums’ ignorance about details of procedures in the respective hospital.

Further points which are worth discussing in addition to the study results relate to organisational and societal aspects. The development of organisational ethics is dependent on the inclusion and commitment of the staff and on the stability of organisational structures [28]. The increasing employment of locums might lead to a situation where it is challenging to establish a shared moral understanding and cooperative practice in hospital organisations. From a societal perspective, German locum physicians currently fulfil the function of a stopgap. They are hired in situations of extreme shortage of staff and help to overcome bottlenecks which result partly from mismanagement and partly occur unexpectedly. In this role, locums are of great importance for the hospitals’ financial outcome and for the reliable provision of patient care. However, from a more holistic perspective, a sustainable management of human resources in the German health care system would be clearly advantageous compared to the current situation in which locums sometimes serve as a sheet anchor to prevent the breakdown of hospital departments. A more stable employment situation of permanent staff is not only cost-effective in the long run, but also forms the precondition for trustful relationships in the physician–patient encounter and within health care teams.

This study is the first explorative investigation of locum physicians in Germany focusing on their perception of ethical duties and complementing the locums’ perspectives with the ones of permanently employed colleagues. Although the study allows for a deepened understanding of the recent phenomenon of locum physicians in German hospitals, the results presented in this paper carry the general limitations of qualitative research, such as non-representativeness. Furthermore, the participants’ self-recruitment might have had an influence on the composition of the sample: The sample might include especially those locums who are not mainly financially driven as we did not provide financial incentives in return for the interviews. In addition, the group of permanently employed physicians was rather small and consisted predominantly of junior doctors. This might have had an influence on this group’s high estimation of locums as unofficial supervisors. The cooperation between experienced locums and junior permanent staff is, however, a typical constellation in German hospitals because the great majority of locums are medical specialists with a long-time clinical experience.

## Conclusions

The increase in locum physicians emerged out of an economisation of the German health care system and the subsequent growing pressure on health care staff and institutions. This development created a new type of professional who deliberately opts out of the system and successfully finds their occupational niche. Ethical duties towards patients, colleagues and society are partially met. However, from the permanent staff’s perspective difficulties remain with respect to close doctor–patient contact. From a societal perspective, the sustainability of using locums as a remedy in times of staff shortage is doubtful. This study provides a first explorative investigation of locum physicians and their relationship to ethical duties, namely the patients, colleagues and society. Further qualitative and quantitative research on locum physicians’ professional practice, including patient perspectives and economic health care system analyses, is needed to better understand the ethical impact of hiring independent contractors in the hospital sector.

## Additional file

Additional file 1: Interview Guide. (DOCX 15 kb)
