# Supplementary material for: Locum physicians’ professional ethos: a qualitative interview study from Germany
Source: BMC Health Serv Res. 2018 May 8;18:333. doi: 10.1186/s12913-018-3118-6 (PMC5941762; doi:10.1186/s12913-018-3118-6)
Supplement: Supplementary file 1 — Interview Guide. (DOCX 15 kb) [file 12913_2018_3118_MOESM1_ESM.docx]

# Additional File 1: Interview guide

| **Topic I: Occupational biography** |
| --- |
| In our project we analyze increasing employment flexibility in the context of a changing healthcare system, which is why we are particularly interested in your professional experience.  Perhaps we can start with your occupational biography: How did it come that you are working as a locum doctor? |
| Why have you decided to work self-employed as a locum doctor? |
| What are the advantages and disadvantages of working as a locum doctor? |
| … |

| **Topic II: Working as a locum doctor** |
| --- |
| **Job description** |
| What is your daily work about? |
| … |
| **Cooperation with colleagues** |
| What are typical conflicts in the cooperation with permanent colleagues?  To which extent can you add new insights and/or contribute your experiences in hospitals? |
| … |
| **Physician-patient relationship** |
| How has your relationship to patients changed since you are locum doctor? |
| … |
| **Cooperation with chief physicians** |
| How is working with chief physicians in hospitals? |
| … |
| **Critical incidents** |
| In everyday clinical practice, unfortunately, from time to time to minor incidents or errors occur.  How do you deal with errors and incidents when you are working as a locum doctor? What consequences do you see when you make an error as a locum doctor?  What are the similarities and differences in the handling of errors compared to permanently employed colleagues?  … |
| **Training and vocational development** |
| How important is training to your activity as a locum doctor?  … |

| **Topic III: Framework conditions & future prospects** |
| --- |
| What do you consider as indispensable qualities for a successful locum doctor? |
| What are the opportunities and risks that may be relevant to your work as a locum doctor? |
| … |
